# RNA secondary structure prediction by conducting multi-class classifications

**DOI:** 10.1016/j.csbj.2025.04.001

**Published:** 2025-04-04

**Authors:** Jiyuan Yang, Kengo Sato, Martin Loza, Sung-Joon Park, Kenta Nakai

**Affiliations:** a Department of Computer Science, the Graduate School of Information Science and Technology, the University of Tokyo, 7-3-1 Hongo, Bunkyo-ku, 113-8656, Tokyo, Japan; b School of Life Science and Technology, Institute of Science Tokyo, 2-12-1-M6-12, Ookayama, Meguro-ku, 152-8550, Tokyo, Japan; c Institute of Medical Science, the University of Tokyo, 4-6-1 Shirokanedai, Minato-ku, 108-8639, Tokyo, Japan

**Keywords:** RNA secondary structure, Multi-class classification, Post-processing, Deep learning

## Abstract

Generating valid predictions of RNA secondary structures is challenging. Several deep learning methods have been developed for predicting RNA secondary structures. However, they commonly adopt post-processing steps to adjust the model output to produce valid predictions, which are complicated and could limit the performance. In this study, we propose a simple method by considering RNA secondary structure prediction as multiple multi-class classifications, which eliminates the need for those complicated post-processing steps. Then, we use this method to train and evaluate our model based on the attention mechanism and the convolutional neural network. Besides, we introduce two additional methods, including data augmentation to further improve the within-RNA-family performance and a method to alleviate the performance drop in the cross-RNA-family evaluation. In summary, we could produce valid predictions and achieve better performance without complex post-processing steps, and we show our additional methods are beneficial to the performance in within-RNA-family and cross-RNA-family evaluations.

## Highlights

• We propose a new method that considers RNA secondary structure prediction as multi-class classifications.
• We show that our method eliminates the need for complex post-processing steps to product valid secondary structures.
• The method is model-agnostic and could be used to train and evaluate models using matrix predictions of structures.

## Graphical abstract

**Figure:**
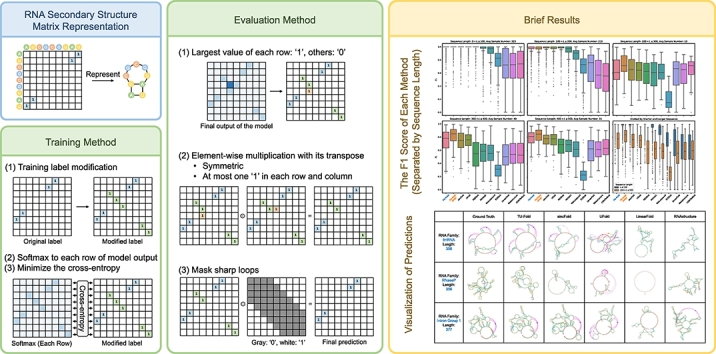

## 1 Introduction

Recent research has revealed that functional non-coding RNAs are essential in various biological processes [1], [2], [3], and the secondary structure is crucial for understanding the function of the RNA molecule [4]. Some early studies experimentally determine the RNA secondary structure by fiber X-ray diffraction [5], optical rotary dispersion [6], and nuclear magnetic resonance [7], while several later approaches use reagents including enzymes and chemicals to probe the RNA secondary structure [8], [9]. However, conducting those experiments is typically difficult, expensive, and time-consuming. Therefore, it is important to develop more efficient methods for RNA secondary structure prediction.

To this end, many studies have tried to computationally predict the RNA secondary structure by using specific algorithms to fold a single RNA sequence [10], [11], [12]. The common approach is energy minimization, which assumes a proper secondary structure has the lowest free energy and predicts the secondary structure by minimizing an energy function. As the energy function comes with various parameters, existing approaches could be roughly divided into two categories based on how those parameters are obtained. Some works use experimentally determined thermodynamic energies of motifs to decide those parameters, such as ViennaRNA [10] and RNAstructure [11], while others learn those parameters from samples of RNA sequences and their secondary structures, like CONTRAfold [12], which leverages the weights of conditional log-linear models learned from those samples. However, the general performance of computational methods is inferior, and there is a need for methods that can predict pseudoknot structures with higher accuracy, which are biologically important in the RNA secondary structure [13], [14].

**Fig. 1:**
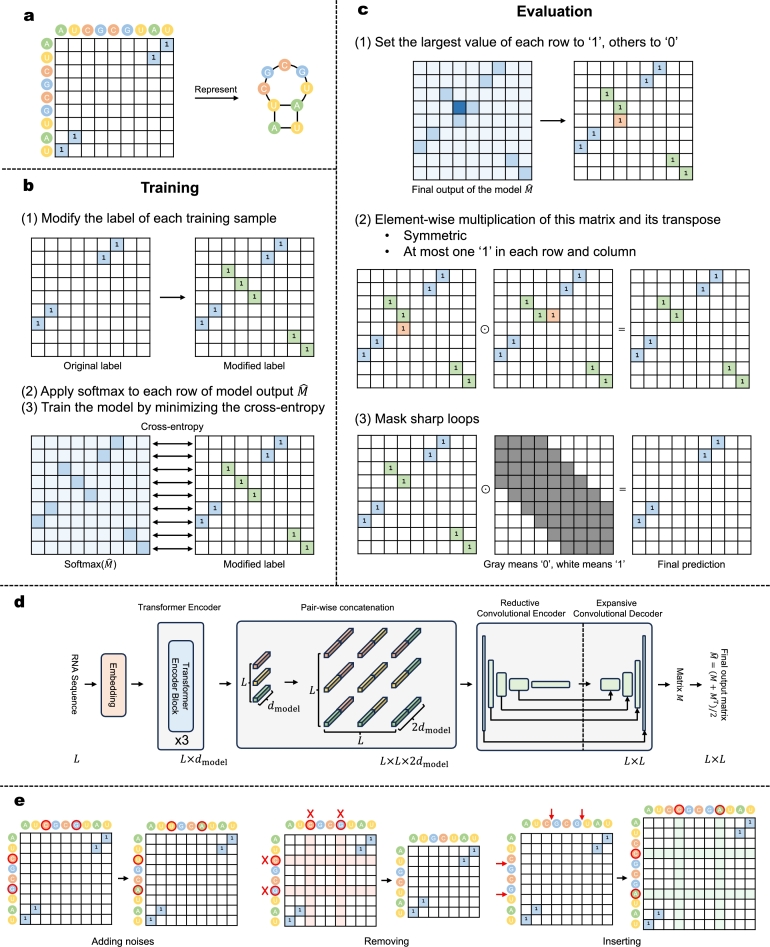
**Overview of methods. a**, an illustration of the matrix representation of the RNA secondary structure. **b**, the training method. We consider each row of the label matrix as a label of multi-class classification and transfer RNA secondary structure prediction to multiple multi-class classifications. **c**, the evaluation method. It is simple to produce valid predictions by several element-wise multiplications. **d**, the overall structure of the model. **e**, illustrations of our data augmentation methods.

Recently, several studies have attempted to use deep learning models for RNA secondary structure prediction [15], [16], [17], [18], [4], [19], [20], [21], [22], [23]. For the RNA sequence with *L* bases, its secondary structure could be represented by an L×L binary matrix, where ‘1’ at the *i*-th row and *j*-th column indicates a base pair between the *i*-th base and the *j*-th base, and ‘0’ represents that two corresponding bases do not form a base pair. Fig. 1a illustrates the matrix representation of the RNA secondary structure. The binary matrix representation makes no assumptions about the RNA secondary structure and could represent any RNA secondary structure, including those with pseudoknots. However, a valid prediction matrix should follow several constraints. Following [17] and [19], we summarize the constraints as follows:

1. Basic constraints:
  (a) the matrix should be binary;
  (b) the matrix should be symmetric;
  (c) there should be at most one ‘1’ in each row and column of the matrix.
2. No sharp loops: for the entry on the *i*-th row and *j*-th column, if |i−j|<4, then this entry should be ‘0’.
3. Possible pairings: the entry on the *i*-th row and *j*-th column could be ‘1’ only if the pair of the *i*-th base and the *j*-th base belongs to canonical pairs (“AU”, “CG”, “GU” pairs) and possible noncanonical pairs.

It is worth noting that the symmetry of the matrix (1b) is one important constraint, since it ensures that one base could pair with at most one base along with constraint (1c). Without constraint (1b), it is possible to set both entry (i,j) and (j,k) to ‘1’ for some k≠i without violating constraint (1c), which means that base *j* pairs with base *i* and base *k* simultaneously. However, constraint (1b) says that if the entry (i,j) is ‘1’, then the entry (j,i) must also be ‘1’. In this case, with constraint (1c), there could not be some entry (j,k) which is also ‘1’ for k≠i. Otherwise, there will be more than one ‘1’ on row *j*, which violates constraint (1c).

Among the constraints above, the most challenging one is that there should be at most one ‘1’ in each row and column of the prediction matrix (1c), since it could not be directly resolved like other constraints by basic matrix operations such as element-wise addition and multiplication. To deal with this constraint, the common paradigm used in existing deep learning methods is to use different post-processing steps to generate the valid prediction matrix based on the output of the model. For example, E2Efold [17] uses a post-processing step based on the primal-dual method to generate the final prediction matrix using the output of a model consisting of the Transformer encoder [24] followed by several convolutional layers. MXfold2 [4] adopts a post-processing step using the Zuker-style dynamic programming [25] to generate the final prediction based on the output of the model composed of the bidirectional long short-term memory (BiLSTM) network [26] and convolutional layers. UFold [19] uses a post-processing step based on linear programming to adjust the output of a model based on the U-Net [27] to produce the valid prediction. The key reason for the necessity of the post-processing steps is that, deep learning methods, such as E2Efold and UFold, consider RNA secondary structure prediction as L×L binary classifications separately, and they overlook the relation among the entries in each row (or column) of the prediction matrix.

In this study, we propose a simple method that avoids the need of complex post-processing steps by considering RNA secondary structure prediction as multiple multi-class classifications. Fig. 1b and Fig. 1c illustrate our training and evaluation method respectively. While training, we modify the label matrix of each training sample by filling the diagonal entries corresponding to zero rows and columns with ‘1’s, which makes it feasible to consider each row (or column) of the label matrix as a label of an *L*-class classification. Therefore, we consider the RNA secondary structure prediction as multiple *L*-class classification tasks. In this case, the number of tasks corresponds to the number of rows (or columns) in the label matrix, which equals *L*. During the evaluation, it is trivial to produce valid predictions by several basic matrix operations illustrated in Fig. 1c. Then, we use this method to train and evaluate our model, named TU-Fold, which consists of a Transformer encoder and a convolutional neural network with the U-Net structure. The model structure of TU-Fold is inspired by E2Efold and UFold, and it is illustrated in Fig. 1d. Besides, we propose two additional methods, namely, data augmentation illustrated in Fig. 1e, to further improve the within-RNA-family performance, and knowledge merge, to alleviate the performance drop in the cross-RNA-family evaluation.

In our experiments, without using complex post-processing steps, TU-Fold achieves even better performance compared with existing deep learning methods. Besides, including training samples generated by using data augmentation further improves the within-RNA-family performance, and knowledge merge helps TU-Fold attain similar cross-RNA-family performance compared with traditional computational methods in a simple and straightforward manner.

## 2 Materials and methods

### 2.1 Training method

The main goal of this study is to propose a simple training and evaluation method for deep learning models which use the matrix representation of the RNA secondary structure as the output, to eliminate the need for comprehensive post-processing steps while generating valid secondary structure predictions.

A formal and comprehensive description of our model is provided in Supplementary Section 2, and detailed illustrations of our model could be found in Supplementary Figs. 5, 6, and 7.

We denote the input RNA sequence length by *L*, and we use an L×L matrix *S* to represent the RNA secondary structure such that

(1) $$S_{ij}=\left\{ \begin{array}{cc} 1\; & \text{if the }i\text{-th base pairs with the }j\text{-th base},\\ 0\; & \text{otherwise} \end{array}\right.$$

where $S_{ij}$ is the entry in the *i*-th row and *j*-th column of the matrix *S*. We denote the output of the model by an L×L matrix *M*, and we make the final output $\overset{ˆ}{M}$ symmetric by taking the average of this matrix and its transpose as follows:

(2) $$\overset{ˆ}{M}=\frac{M+M^{T}}{2}.$$

While training, we first modify the label of each training sample by filling the diagonal entries corresponding to zero rows with ‘1’s. More rigorously, if we denote the modified label matrix by $\overset{ˆ}{S}$, then

(3) $${\overset{ˆ}{S}}_{ij}=\left\{ \begin{array}{cc} S_{ij}\; & \text{for }i\neq j,\\ 1-\sum_{i}S_{ij}\; & \text{for }i=j. \end{array}\right.$$

The benefits of this modification for the labels of training samples are three-fold. First, there is exactly one ‘1’ in each row (or column) of the label matrix, which makes it feasible to consider each row (or column) as a label of an *L*-class classification. Second, the modified label matrix is still symmetric. Third, there is no modification to the model. After this modification, each row (or column) of the label matrix could be considered as a label of an *L*-class classification, and the RNA secondary structure prediction task could be therefore transferred to *L L*-class classifications.

We provide a detailed discussion in Supplementary Section 3 to demonstrate that it is sufficient to consider either rows or columns as *L*-class classifications to train the model, and we show that they are equivalent. Therefore, we consider each row of the label matrix as a label of an *L*-class classification in the following.

We apply softmax to each row of $\overset{ˆ}{M}$, and we denote the result by an L×L matrix $P^{r}$. Then, we train the model by minimizing the cross-entropy between each row of $P^{r}$ and $\overset{ˆ}{S}$. As the number of bases forming base pairs and the number of bases not forming base pairs are often unbalanced, instead of using the normal average cross-entropy, we introduce a weight for each cross-entropy, and we use the weighted average cross-entropy as the optimization target. We denote the shape of the prediction of a batch of RNA sequences by (b,L,L), where *b* is the batch size. We say that a row of the prediction matrix and the corresponding row of the label matrix form a sub-sample. In each batch, the number of sub-samples is *bL*. We denote the cross-entropy of the *i*-th sub-sample by $\ell_{i}$, where i∈{1,2,…,bL}. Then, we denote the set of the indices of sub-samples whose bases do not form base pairs by **D**, and the size of **D** by |D|. Using the notations above, the optimization target *ℓ* is calculated by

(4) $$\ell =\frac{1}{bL}\left(\sum_{i\notin D}\ell_{i}+\frac{bL-|D|}{\left|D\right|}\sum_{i\in D}\ell_{i}\right),$$

and we train the model by minimizing *ℓ*.

### 2.2 Evaluation method

During the evaluation, we first set the largest value of each row of the model output $\overset{ˆ}{M}$ to ‘1’ and others to ‘0’, and we denote the result by an L×L matrix ${\overset{ˆ}{P}}^{r}$. Although $\overset{ˆ}{M}$ is symmetric, ${\overset{ˆ}{P}}^{r}$ is by no means guaranteed to be symmetric. Therefore, we have to further process ${\overset{ˆ}{P}}^{r}$ to produce a symmetric prediction matrix for the RNA secondary structure.

As ${\overset{ˆ}{P}}^{r}$ is a binary matrix, to make it symmetric, during the evaluation, we calculate the element-wise product of ${\overset{ˆ}{P}}^{r}$ and its transpose denoted by ${\overset{ˆ}{P}}^{r}⊙{\left({\overset{ˆ}{P}}^{r}\right)}^{T}$, where ⊙ indicates the element-wise product.

The result of the element-wise product is not only binary and symmetric, but there is also at most one ‘1’ in each row and column. We give a brief explanation about this. We know that each row of ${\overset{ˆ}{P}}^{r}$ contains exactly one ‘1’, but there could be multiple ‘1’s in a column of ${\overset{ˆ}{P}}^{r}$. Suppose that ${\overset{ˆ}{P}}_{ji}^{r}={\overset{ˆ}{P}}_{ki}^{r}=1$, where j≠k. As each row of ${\overset{ˆ}{P}}^{r}$ contains one ‘1’, at most one of ${\overset{ˆ}{P}}_{ij}^{r}$ and ${\overset{ˆ}{P}}_{ik}^{r}$ could be ‘1’, thereby at most one of ${\overset{ˆ}{P}}_{ji}^{r}{\overset{ˆ}{P}}_{ij}^{r}$ and ${\overset{ˆ}{P}}_{ki}^{r}{\overset{ˆ}{P}}_{ik}^{c}$ could be ‘1’. This suggests that ${\overset{ˆ}{P}}^{r}⊙{\left({\overset{ˆ}{P}}^{r}\right)}^{T}$ contains at most one ‘1’ in each row and column.

We do not apply any mask for the “possible pairings” constraint. First, there are not only canonical pairs but also non-canonical pairs, which have important roles [28], and recent research suggests the existence of even more diverse kinds of pairs [29]. The diversity of pairs makes it hard to decide whether two bases could form a base pair or not. Second, the samples of RNA secondary structures in the dataset may not be completely accurate and may include noises. Instead of merely focusing on the correctness of the prediction of each pair of bases, the overall structure indicated by the prediction is more important. Therefore, using masks for the “possible pairings” constraint could make the method rigid and weak to noise, and we let the model to learn this constraint from the data by itself.

During the evaluation, we only apply a mask for the “no sharp loops” constraint. We denote the mask matrix by *Z*, such that

(5) $$Z_{ij}=\left\{ \begin{array}{cc} 0\; & \text{if }|i-j|<4,\\ 1\; & \text{otherwise.} \end{array}\right.$$

Then, the final prediction $\overset{ˆ}{P}$ is calculated by

(6) $$\overset{ˆ}{P}={\overset{ˆ}{P}}^{r}⊙{\left({\overset{ˆ}{P}}^{r}\right)}^{T}⊙Z.$$

While training the model, we let it learn to predict the corresponding diagonal entry of a base in the prediction matrix to be ‘1’ if this base does not form a base pair. In other words, for bases not forming base pairs, we let the model to predict that each of those bases “pairs with itself”. During the evaluation, we use the matrix *Z* to mask entries suggesting sharp loops, which indeed include those diagonal entries. Therefore, a base predicted to “pair with itself” is finally predicted to form no base pair during the evaluation.

### 2.3 Data augmentation

We propose three simple data augmentation methods for RNA samples to generate more training samples to further improve the within-RNA-family performance of the model.

Data augmentation methods based on random modifications are widely used for improving the performance of deep learning models. However, compared with the data augmentation of other kinds of data, it is hard to assign an accurate secondary structure to each randomly augmented RNA sequence based on the original structure, as it is infeasible to evaluate how the modification to the RNA sequence influences its secondary structure. Since large random modifications to the RNA sequence lead to large influence on the RNA secondary structure, only small random modifications are practical. Besides, we only modify bases not forming base pairs to alleviate such kind of influence.

For each sample, we conduct three kinds of augmentation, including adding noises, removing, and inserting. Fig. 1e is an illustration of our data augmentation methods. For an RNA sequence **x** and its secondary structure *S*, we denote the set of bases forming no base pairs by **B**, and we choose small percentages of three kinds of augmentation denoted by $p_{1},p_{2},p_{3}$ respectively. As for adding noises, we select $p_{1}$ percentage of bases from **B**, and change each of them to another different base. The original structure *S* is used as the structure of the augmented sequence. As for removing, we select $p_{2}$ percentage of bases from **B**, and remove them from the RNA sequence, followed by adding noise. The structure of the augmented sequence is obtained by removing the rows and columns of *S* corresponding to the removed bases. As for inserting, we select $p_{3}$ percentage of positions, insert one random base to each of those positions, followed by adding noise. Then, its structure is obtained by inserting zero rows and columns into *S* correspondingly.

### 2.4 Knowledge merge

Deep learning typically assumes that samples from the training dataset and the test dataset are identically distributed [30]. However, in the cross-RNA-family evaluation, since the samples in the test dataset come from a different RNA family not covered by the training dataset, the training dataset and the test dataset are not identically distributed at all, and the cross-RNA-family evaluation is not a typical task in the study of deep learning. In this task, exclusively data-driven deep learning methods usually have inferior performance during the evaluation due to the large distribution gap between the training data and test data.

In this study, we propose “knowledge merge”, attempting to alleviate the performance drop in the cross-RNA-family evaluation to some extent, which inspired by knowledge distillation [31] and [19].

The key idea is to use randomly synthesized RNA sequences and predictions of their secondary structures produced by computational methods as supplementary training samples to train the model. Computational methods directly fold the RNA sequence by using specific algorithms, and they do not rely on training samples like deep learning methods. As a result, computational methods can keep the performance when testing on different RNA families. Therefore, we leverage computational methods to improve the performance of our deep learning model when testing on RNA families not covered by the training dataset. Different from [19], since we use totally synthesized RNA sequences, we do not rely on any real RNA samples, and our method is more general and straightforward.

We first describe the knowledge of an RNA secondary structure prediction method. We denote the set of all possible RNA sequences by **X** containing every string composed of ‘A’, ‘U’, ‘C’, and ‘G’ whose length ranges from the lower bound $L_{1}$ to the upper bound $L_{2}$. We then denote the set of all possible RNA secondary structure matrices by **S**. We say the knowledge of an RNA secondary structure prediction method is a mapping f:X↦S associating each element of **X** with one element of **S**.

In our method, we make a statistical approximation of the knowledge of a computational method *f*. Specifically, each time we randomly select a sequence from **X**, and we use a computational method to predict its secondary structure. We repeat this process to generate a large set of “sequence-prediction” pairs. We consider this set as an approximation of the knowledge *f* of the “teacher”, and we use those synthesized samples as supplementary training samples to train the model to merge this knowledge to a deep learning model.

### 2.5 Experimental settings

Our experiments are based on the RNA8F dataset containing RNA sequences (L≤500) and secondary structures from 8 RNA families. The RNA8F dataset is constructed from four public datasets: RNAStrAlign [32], ArchiveII [33], bpRNA [34], and CRW2 [35]. Since those raw datasets are differently organized and contain RNA samples in various formats, they are first cleaned and processed such that

1. the RNA sequence is a string composed of ‘A’, ‘U’, ‘C’, and ‘G’;
2. the length of each sequence is less than 500;
3. the RNA secondary structure does not contain sharp loops;
4. invalid RNA samples are removed;
5. samples are named in a consistent format;
6. the RNA sequence is stored in the FASTA format, and the RNA secondary structure is stored in the CT format.

Then, we use the CD-HIT program [36], [37] to remove similar samples with threshold 0.9, to obtain sufficient samples for the training and evaluation. For the convenience of Python-based deep learning models, we further use NumPy arrays to store the sequence and secondary structure, which are illustrated in Supplementary Fig. 4a. Following [17], we split it into train, valid, and test sets, and a detailed summary of this dataset is available in Table 1. We also provide the distribution of the length of the RNA sequence from each RNA family in Supplementary Fig. 4b. Three random train-valid-test partitions are used for the evaluation of the performance.

**Table 1:** **Summary of the RNA8F dataset.** Each entry is the sample number of the corresponding RNA family in the train, valid, and test set.

	50<L≤150			150<L≤500		
RNA Family	Train	Valid	Test	Train	Valid	Test
16S rRNA	3	0	0	14	2	1
5S rRNA	1636	204	204	0	0	0
Intron Group 1	0	0	0	376	47	46
RNaseP	1	0	0	100	13	12
SRP	105	13	13	92	12	11
Telomerase	0	0	0	23	3	2
tRNA	2528	316	316	0	0	0
tmRNA	1	0	0	184	23	22

Total	4274	533	533	789	100	94

Deep learning methods evaluated in our experiments include TU-Fold, sincFold [23], REDfold [22], UFold [19], MXfold2 [4], RNA-state-inf [18], and E2Efold [17]. Several traditional computational methods including LinearFold [38], RNAstructure [11], and CONTRAfold [12], are also evaluated in the experiments. For RNAstructure, we use the ProbKnot [39] program in RNAstructure package. For LinearFold, we choose LinearFold-C using CONTRAfold parameters, which is the default setting of the LinearFold program. Since CONTRAfold, RNAstructure, and LinearFold are not trainable, following [17], we directly use their programs to generate predictions of secondary structures of RNA sequences from the test set.

In the experiments, the F1 score and the interaction network fidelity (INF) score [40] are evaluated. The F1 score is calculated using the precision (P) and recall (R), which are defined as follows,

(7) $$P=\frac{TP}{TP+FP},\;R=\frac{TP}{TP+FN},\;F1=\frac{2PR}{P+R},$$

and the INF score is defined as

(8) $$INF=\frac{TP\times TN-FP\times FN}{\sqrt{\left(TP+FP)(TP+FN)(TN+FP)(TN+FN\right)}},$$

where TP, FP, TN, FN are the numbers of true positives, false positives, true negatives, false negatives respectively. The INF score is also a metric used for the evaluation of RNA secondary structure predictions and is calculated identically to the Matthews correlation coefficient (MCC).

In the experiment of data augmentation, using the notations in Section 2.3, we intuitively set the augmentation percentages $p_{1}=p_{2}=p_{3}=10\text{\%}$. For each longer RNA sample (150<L≤500), we apply adding noise, removing, and inserting twice, to generate six augmented samples.

In the experiment of knowledge merge, we choose LinearFold [38] to generate synthesized samples, due to its relatively small time complexity, which makes it possible to obtain abundant “sequence-prediction” pairs in feasible time. As for the length of the synthesized RNA sequence, using the notations in Section 2.4, we set the lower bound $L_{1}=50$ and the upper bound $L_{2}=500$. For each synthesized sample, we first randomly select a sequence length bounded by $L_{1}$ and $L_{2}$, then we randomly generate an array composed of ‘1’, ‘2’, ‘3’, and ‘4’, suggesting ‘A’, ‘U’, ‘C’, and ‘G’ respectively. This synthesized sequence is then passed to LinearFold, and the prediction of LinearFold is used as the label of this synthesized sequence. Following the procedure above, we randomly synthesize 30,000 RNA samples and use those synthesized samples as supplementary training samples.

## 3 Results

### 3.1 TU-fold outperforms existing methods without using post-processing steps

**Fig. 2:**
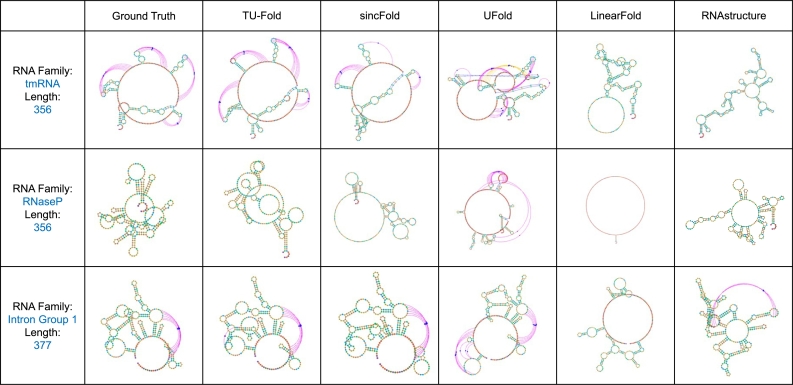
**Visualization of Structures.** We select and visualize several RNA secondary structure predictions. The colored curves in each visualization indicate the base pairs in pseudoknots.

In this section, we provide an overview of the main results, followed by two detailed analyses from the perspectives of the RNA sequence length and the RNA family. The goal of our study is to propose a simple and effective method for training and evaluating deep learning models without using comprehensive post-processing steps while producing valid secondary structure predictions. Therefore, the main concern of our work is the within-RNA-family performance. The main results of the F1 score and INF score of each method are reported in Table 2, and the precision and recall are reported in Table 3 for reference. Besides, Fig. 2 gives some examples of RNA secondary structure predictions, which are plotted using the “jViz.Rna” software [41].

**Table 2:** **Main results: the F1 score and the INF score of each method.** The largest score in each column is highlighted in bold. The score with an underline is the second largest one in each column. The numbers on the left and right of the ‘±’ suggest the mean and the standard deviation of this score when training and evaluating the model using 3 folds of the dataset.

	Overall		50<L≤150		150<L≤500	
Method	F1	INF	F1	INF	F1	INF
sincFold	0.936±0.002	0.938±0.002	0.974±0.003	0.975±0.003	0.718±0.006	0.729±0.007
UFold	0.938±0.004	0.938±0.004	**0.976**±0.003	**0.976**±0.003	0.719±0.030	0.720±0.030
REDfold	0.916±0.003	0.916±0.003	0.975±0.001	0.976±0.001	0.581±0.014	0.582±0.014
MXfold2	0.894±0.004	0.894±0.004	0.947±0.006	0.947±0.005	0.596±0.005	0.597±0.005
RNA-state-inf	0.844±0.002	0.844±0.002	0.891±0.002	0.891±0.002	0.579±0.022	0.580±0.022
E2Efold	0.673±0.005	0.675±0.005	0.747±0.004	0.749±0.005	0.253±0.015	0.256±0.014
LinearFold	0.668±0.002	0.670±0.001	0.688±0.001	0.690±0.001	0.554±0.016	0.556±0.015
RNAstructure	0.628±0.008	0.628±0.008	0.639±0.007	0.638±0.007	0.570±0.022	0.571±0.022
CONTRAfold	0.626±0.015	0.626±0.015	0.634±0.017	0.633±0.017	0.584±0.008	0.586±0.007

TU-Fold	0.940±0.001	0.941±0.001	0.976±0.001	0.976±0.001	0.735±0.012	0.741±0.013
TU-Fold (aug)	**0.947**±0.002	**0.947**±0.002	0.973±0.003	0.973±0.003	**0.797**±0.016	**0.802**±0.015

**Table 3:** **The P and R of each method.** The largest score in each column is highlighted in bold. The score with an underline is the second largest one in each column. The numbers on the left and right of the ‘±’ suggest the mean and the standard deviation of this score when training and evaluating the model using 3 folds of the dataset.

	Overall		50<L≤150		150<L≤500	
Method	P	R	P	R	P	R
sincFold	**0.960**±0.002	0.920±0.004	**0.982**±0.002	0.969±0.004	0.840±0.024	0.640±0.007
UFold	0.936±0.007	**0.941**±0.002	0.974±0.004	**0.980**±0.002	0.720±0.048	0.725±0.016
REDfold	0.917±0.002	0.917±0.004	0.975±0.001	0.977±0.002	0.594±0.017	0.574±0.013
MXfold2	0.891±0.004	0.899±0.004	0.943±0.006	0.952±0.005	0.599±0.005	0.598±0.005
RNA-state-inf	0.831±0.002	0.860±0.001	0.879±0.002	0.905±0.003	0.559±0.021	0.605±0.023
E2Efold	0.631±0.018	0.733±0.021	0.703±0.018	0.808±0.025	0.220±0.018	0.307±0.016
LinearFold	0.684±0.005	0.668±0.004	0.702±0.006	0.691±0.003	0.581±0.015	0.539±0.015
RNAstructure	0.602±0.007	0.662±0.008	0.612±0.008	0.672±0.006	0.546±0.021	0.601±0.026
CONTRAfold	0.608±0.015	0.653±0.015	0.614±0.017	0.661±0.017	0.571±0.002	0.606±0.011

TU-Fold	0.954±0.003	0.931±0.001	0.978±0.003	0.975±0.002	0.821±0.020	0.677±0.008
TU-Fold (aug)	0.958±0.004	0.940±0.001	0.974±0.005	0.973±0.002	**0.864**±0.012	**0.752**±0.017

Using the simple training and evaluation method by conducting multi-class classifications, TU-Fold outperforms deep learning models including sincFold, UFold, REDfold, MXfold2, RNA-state-inf, and E2Efold with 0.4%, 0.2%, 2.4%, 4.6%, 9.6%, and 26.7% improvements in the mean F1 score respectively. Compared to previous methods such as UFold and E2Efold, our method could achieve similar or even better performance, without complex post-processing steps while generating valid predictions, which suggests that the method of transferring RNA secondary structure prediction task to multiple multi-class classifications could be a potential replacement of the post-processing steps used in previous studies.

While the overall results above demonstrate the effectiveness of our method to some extent, it is worth emphasizing that samples in the dataset are highly biased. Specifically, the sample number of shorter RNA sequences (50<L≤150) is much larger than the one of longer RNA sequences (150<L≤500), and the sample number of RNA sequences from each RNA family varies significantly as well. Therefore, apart from overall average scores, we further analyze the performance of each method when testing on RNA sequences of different lengths and RNA sequences from different RNA families.

To analyze the performance of different methods from the perspective of the RNA sequence length, we calculate the average scores of the predictions of shorter RNA sequences and longer RNA sequences separately. Here, we use 150 as the sequence length for splitting shorter and longer sequences, one threshold suggested in [42]. We would like to make it clear that in the training of the model, all samples include both of those shorter ones and longer ones are used for training the models, and this threshold is chosen for the convenience of presentation.

**Fig. 3:**
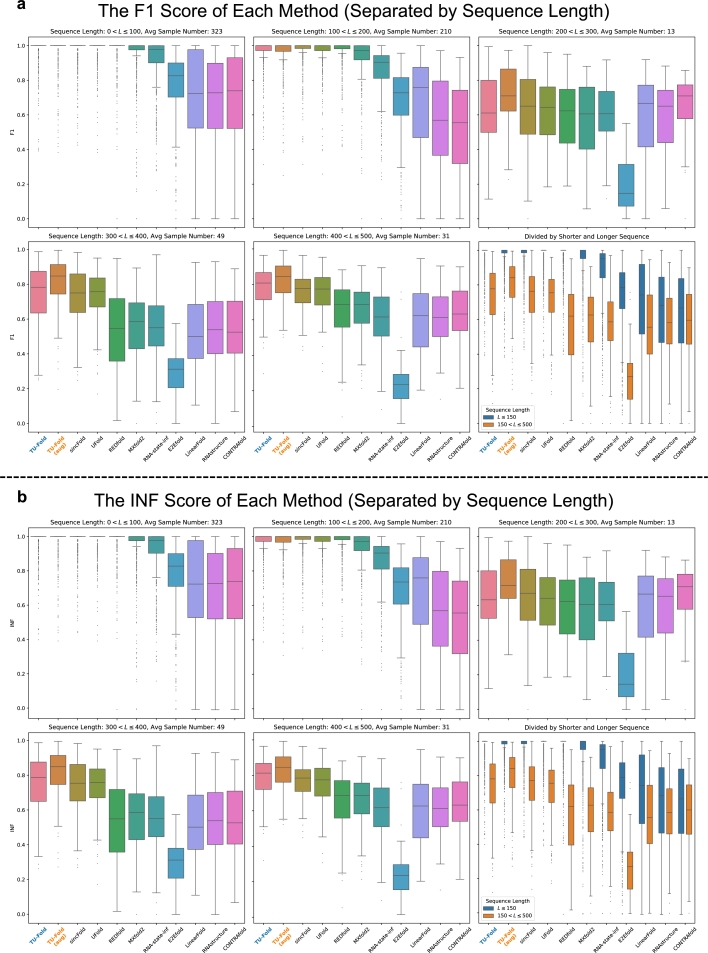
**Results of the F1 score and the INF score Separated by Sequence Length. a**, the box plot of the F1 score of each method. The first 5 plots (plots except the one on the right bottom corner) illustrate the F1 scores of test samples separated by an interval of sequence length 100. The last one is an overview of the F1 scores of shorter and longer test samples. **b**, the barplot of the INF score of each method.

The results are reported in the “50<L≤150” column and “150<L≤500” column of Table 2 respectively. Fig. 3 provides a more fine-grained illustration, which contains box plots of the F1 and INF scores of test samples separated by intervals of length 100.

Most methods achieve much better scores when testing on shorter RNA sequences, and all of sincFold, UFold, REDfold, MXfold2, and TU-Fold obtain average F1 scores larger than 0.9. However, when testing on longer RNA sequences, the difference among the performance of those methods becomes more evident, and the results show that, TU-Fold outperforms sincFold, UFold, REDfold, MXfold2 by 1.7%, 1.6%, 15.4%, 13.9% with regard to the F1 score, and 1.2%, 2.1%, 15.9%, 14.4% with regard to the INF score respectively.

**Fig. 4:**
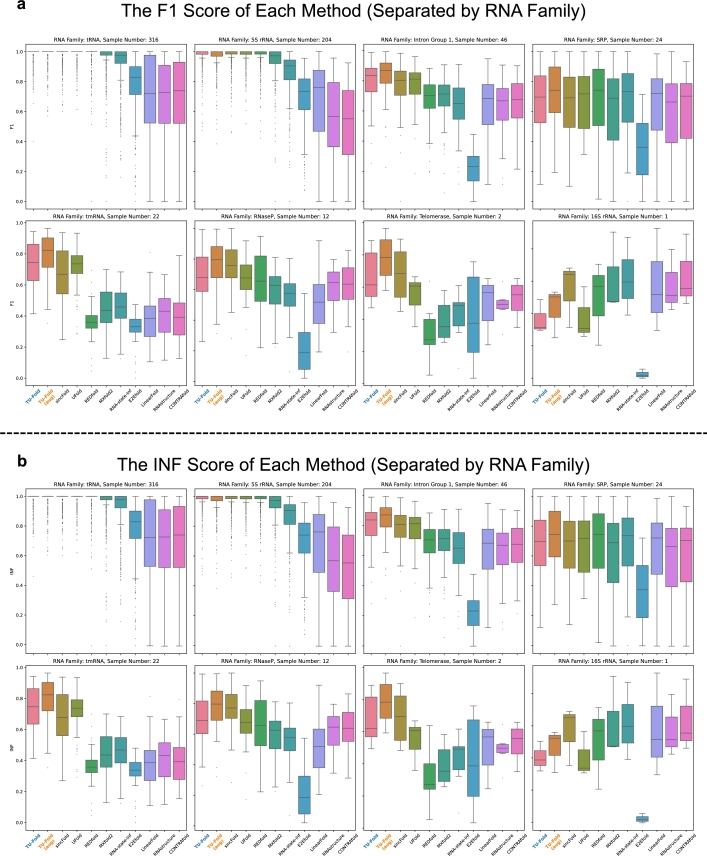
**Results of the F1 score and the INF score Separated by RNA Family. a**, the box plot of the F1 score of each method. Each plot illustrates the F1 scores of samples from the corresponding RNA family. **b**, the box plot of the INF score of each method.

Besides, Fig. 4 provides an illustration of the performance of different methods from the perspective of the RNA family. It is worth noting that the results of the scores are somewhat analogous to those when testing on RNA sequences with different length, since 5S rRNA and tRNA mainly contain shorter RNA sequences, and the sample numbers of 5S rRNA and tRNA are much larger compared to other 6 RNA families, which mainly contain longer RNA sequences. As for those 6 RNA families, due to the longer length and smaller sample number, the performance of most methods becomes inferior. Nevertheless, our method still achieves better performance when testing on those RNA families compared with other methods, especially E2Efold, MXfold2, and UFold.

### 3.2 Data augmentation further improves the performance

As discussed in the previous section, our study is aim to propose a method for the training and evaluation of deep learning models without comprehensive post-processing steps while producing valid predictions, and one typical evaluation of its effectiveness is the within-RNA-family performance. In this section, we analyze the effectiveness of data augmentation methods, by which we attempt to deal with the issue of the limited number of training samples to further improve the performance.

In the experiments, we do not augment shorter RNA samples (50<L≤150), which mainly contains samples from 5S rRNA and tRNA, as the number of them is relatively sufficient. We only apply data augmentation methods to longer RNA samples (150<L≤500), which consists of RNA samples from 6 RNA families other than 5S rRNA and tRNA.

The main results of TU-Fold after using data augmentation methods are reported in the “TU-Fold (aug)” row of Table 2, and the corresponding box plots “TU-Fold (aug)” in Fig. 3 and Fig. 4 are the results of the F1 and INF score from the perspective of the sequence length and the RNA family.

The results demonstrate that training with augmented samples further improves the performance. While the scores of the predictions of RNA sequences from 5S rRNA and tRNA are almost unchanged, as we do not apply data augmentation to those two RNA families, the performance when testing on other 6 RNA families containing augmented training samples becomes even better, and we observe a 6.2% improvement in the F1 score and a 6.1% improvement in the INF score.

Besides, we conduct experiments to show that simply increasing the sample number of longer RNA sequences by duplicating is not beneficial to the performance, suggesting the effectiveness of the data augmentation methods, and the results are reported in Supplementary Fig. 1.

### 3.3 Knowledge merge alleviates the performance drop in the cross-RNA-family evaluation

As discussed in Section 2.4, without any further regularization based on specialized knowledge, methods exclusively based on deep learning have inferior performance in the cross-RNA-family evaluation [4], [20]. While it is not a typical evaluation task for deep learning models and not suited to exclusively data-driven methods, we attempt to develop a simple method to alleviate the performance drop of deep learning methods in the cross-RNA-family evaluation to some extent.

**Fig. 5:**
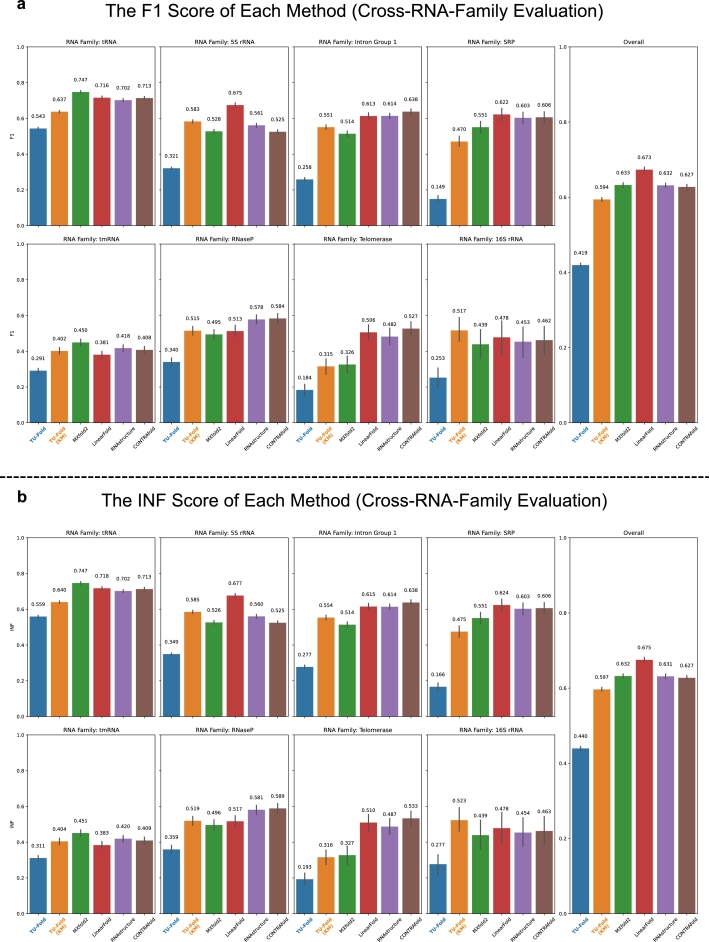
**Results of the cross-RNA-family evaluation. a**, the F1 scores of each RNA family and the overall average F1 score in the cross-RNA-family evaluation. **b**, the INF scores of each RNA family and the overall average INF score in the cross-RNA-family evaluation.

The results of the cross-RNA-family evaluation are reported in Fig. 5, where “TU-Fold” and “TU-Fold (KM)” indicate the results before and after applying knowledge merge respectively. Each subplot in Fig. 5 contains the F1 or the INF score of each method when training on the samples from other 7 RNA families and testing on the samples from the corresponding RNA family of this subplot, and the “Overall” subplot indicates the average performance. For comparison, we also report the F1 and the INF scores of traditional computational methods including CONTRAfold [12], RNAstructure [11], and LinearFold [38]. As these traditional computational methods are not trainable, we directly evaluate their performance when testing on each RNA family using their programs. Besides, we also report the F1 and the INF scores of MXfold2 by directly conducting the cross-RNA-family evaluation without using synthesized samples, since it includes a thermodynamic regularization, an approach based on the specialist knowledge of the RNA secondary structure, to improve the cross-RNA-family performance.

After applying knowledge merge, the performance drop is alleviated, which indicates that knowledge merge is beneficial to the model when testing on RNA families not covered by the training dataset and it helps the model achieve similar performance to traditional computational methods. Besides, since we use the matrix representation of the RNA secondary structure, as introduced in Section 1, we could predict structures with pseudoknots compared with traditional methods.

To quantitatively illustrate that the knowledge of LinearFold is merged to our model, for the prediction of each base in an RNA sequence, we use the ratio of same predictions of bases in each sequence to show the correlation between the predictions between our model and LinearFold. Compared to the model without using knowledge merge, after applying knowledge merge, the average same prediction ratio increased from 0.568 to 0.731, and the corresponding box plots could be found in Supplementary Fig. 2.

While reducing the performance drop, one major limitation of knowledge merge is that the performance is bounded by the “teacher” method, since the model is just following the predictions of the teacher methods. Fig. 5 also shows that TU-Fold trained with knowledge merge could not obtain F1 scores better than LinearFold in most cases. As a result, knowledge merge could only be used to alleviate the performance drop to some extent, compared to the performance without using knowledge merge in the cross-RNA-family evaluation.

## 4 Discussion

Our work presents a simple and effective deep learning method for RNA secondary structure prediction. The main contribution of this study is a novel method for training and evaluating the deep learning model by considering RNA secondary structure prediction as multiple multi-class classifications. By filling the diagonal entries corresponding to zero rows and columns of the label matrix of each training sample with ‘1’s, we convert the RNA secondary structure prediction task to multiple classification tasks and solve them by common operations such as the softmax and the cross-entropy used in most classification tasks. We use this method to train and evaluate TU-Fold, a model composed of a Transformer encoder and a convolutional neural network using the U-Net structure. We show that without using any complicated post-processing steps, TU-Fold could generate valid prediction of the RNA secondary structure, and it outperforms existing methods such as E2Efold, MXfold2, and UFold. The method that predicting the RNA secondary structure by conducting multi-class classifications makes it simpler to produce valid and accurate matrix prediction of the RNA secondary structure. It is also worth emphasizing that this method is model-agnostic, and it is possible to apply this method to any model using the matrix prediction of the RNA secondary structure.

Besides, to further improve the within-RNA-family performance and alleviate the performance drop in the cross-RNA-family evaluation, we propose two additional methods, namely, data augmentation and knowledge merge. By applying data augmentation methods including adding noise, removing, and inserting, we achieve even better within-RNA-family performance. By using knowledge merge, in the cross-RNA-family evaluation, we achieve similar performance to MXfold2, CONTRAfold, RNAstructure, and LinearFold.

There are three main limitations of this work. First, as for the model, the maximum length of the input RNA sequence is limited. As the length of the input RNA sequence grows, both the size of attention matrices in the Transformer encoder and the size of feature maps in the convolutional neural network increase quadratically. As a result, the acceptable length of the input RNA sequence is limited due to the limitation of the computing resource. Second, as for the data augmentation, it is hard to assign accurate structures to augmented RNA sequences using simple random augmentations, and developing generative models for generating RNA samples based on existing samples may be a future direction. Third, as for knowledge merge, it could not be used to improve the performance of the model when testing on RNA families covered and not covered by the training dataset simultaneously. The main reason is that the label of each synthesized sample is the prediction from a computational method, which is not accurate and could be harmful to the model when testing on RNA families covered by the training dataset. Consequently, knowledge merge could not be considered as a further step towards improving the overall performance of the model, and it could only be used to deal with RNA families not covered by the training dataset. As a result, the model trained with data augmentation denoted by “TU-Fold (aug)” is recommended when conducting within-RNA-family predictions, and the model trained with knowledge merge denoted by “TU-Fold (KM)” should be used to predict the secondary structures of sequences from RNA families not covered by the training dataset.

## Funding

10.13039/501100001691Japan Society for the Promotion of Science [22K06189].

## CRediT authorship contribution statement

**Jiyuan Yang:** Writing – original draft, Software, Methodology, Conceptualization. **Kengo Sato:** Writing – review & editing, Supervision. **Martin Loza:** Writing – review & editing, Supervision. **Sung-Joon Park:** Writing – review & editing, Supervision, Resources. **Kenta Nakai:** Writing – review & editing, Supervision, Resources, Funding acquisition.

## Declaration of Competing Interest

The authors declare that they have no known competing financial interests or personal relationships that could have appeared to influence the work reported in this paper.

## Data Availability

The source code is available at GitHub https://github.com/ygjiyn/tu_fold, and the datasets and model weights could be found at the release page of our GitHub repository https://github.com/ygjiyn/tu_fold/releases/tag/v0.0.1. Besides, for the convenience of using our model, we build a whl package of our project, which is also available at the release page of our GitHub repository and could be installed using pip in a standard manner.

## Appendix A Supplementary material

MMC Supplementary figures related to the experiments, a detailed description of the model structure, and an in-depth discussion about the training method. MMC
