# Supplementary material for: RNA secondary structure prediction by conducting multi-class classifications
Source: Comput Struct Biotechnol J. 2025 Apr 4;27:1449–59. doi: 10.1016/j.csbj.2025.04.001 (PMC12008525; doi:10.1016/j.csbj.2025.04.001)
Supplement: MMC — Supplementary figures related to the experiments, a detailed description of the model structure, and an in-depth discussion about the training method. [file mmc1.pdf]

Supplementary Material
RNA Secondary Structure Prediction by Conducting Multi-Class Classifications

Jiyuan Yang[1], Kengo Sato[2], Martin Loza[3], Sung-Joon Park[1,3], and Kenta Nakai[1,3,*]

[1]Department of Computer Science, the Graduate School of Information Science and Technology, the University of Tokyo, 7-3-1 Hongo, Bunkyo-ku, 113-8656, Tokyo, Japan
[2]School of Life Science and Technology, Tokyo Institute of Technology, 2-12-1-M6-12, Ookayama, Meguro-ku, 152-8550, Tokyo, Japan
[3]Institute of Medical Science, the University of Tokyo, 4-6-1 Shirokanedai, Minato-ku, 108-8639, Tokyo, Japan
[*]Corresponding author. E-mail: knakai@ims.u-tokyo.ac.jp

Contents

1 Supplementary Tables and Figures **1**

2 Supplement: Details of the Model Structure **6**
2.1 Input Embedding . . . 6
2.2 Transformer Encoder . . . 6
2.3 Pair-wise Concatenation . . . 8
2.4 U-Net Encoder . . . 8
2.5 U-Net Decoder . . . 9

3 Supplement: the Optimization of the Model **10**

1 Supplementary Tables and Figures

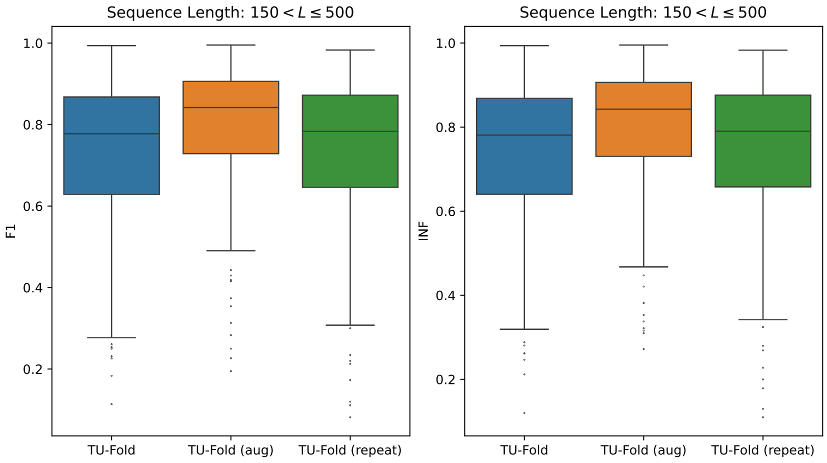

Supplementary Figure 1: **Comparing duplication and data augmentation.** Each box plot of "TU-Fold (repeat)" suggests the F1 and INF score when simply duplicating longer RNA sequences instead of using data augmentation methods. Compared to the results of "TU-Fold" and "TU-Fold (aug)", this kind of duplication is not beneficial to the performance.

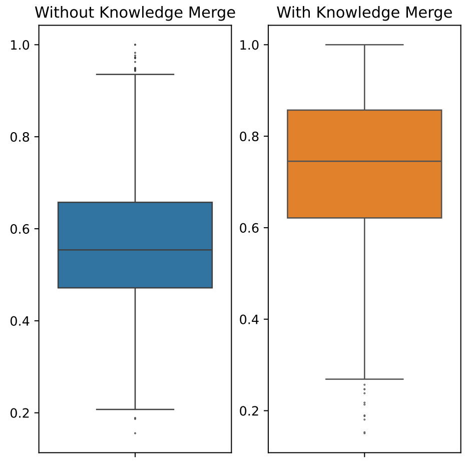

Supplementary Figure 2: **The correlation between the prediction of ours and LinearFold in the cross-RNA-family evaluation.** After applying knowledge merge, the prediction of our model shows higher correlation to the teacher model LinearFold, which suggests that knowledge merge makes our model learn the knowledge from LinearFold.

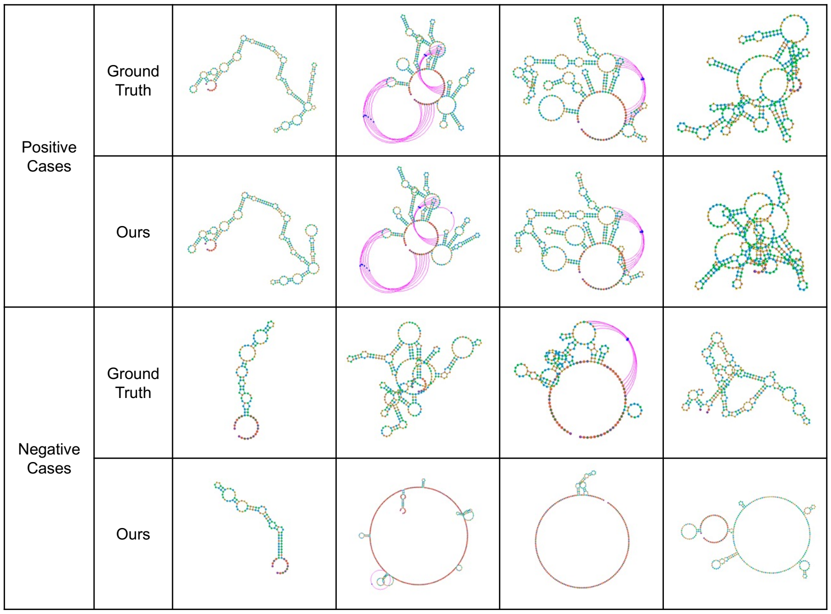

Supplementary Figure 3: **Positve cases and negative cases of our predictions.** Visualizations of some relatively good (positive case) and bad (negative case) predictions of our model.

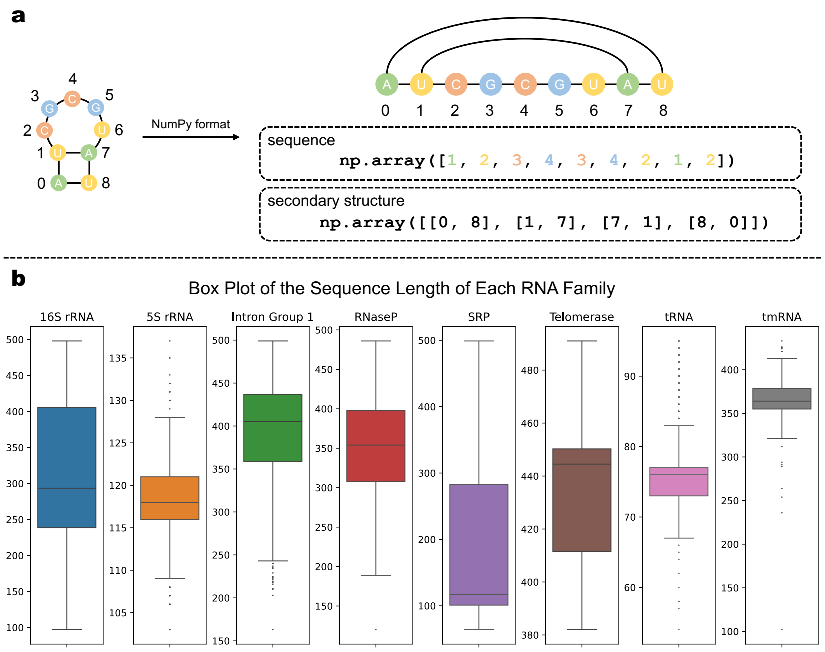

Supplementary Figure 4: **Further information about the dataset.** **a**, the NumPy-based format used in the RNA8F dataset for storing the RNA sequence and the RNA secondary structure. **b**, the box plot of the sequence length in each RNA family.

2 Supplement: Details of the Model Structure

We use the Transformer encoder [1] followed by a U-Net [2] to produce the matrix prediction of the secondary structure.

2.1 Input Embedding

We denote the maximum length of the input RNA sequence by L. For an RNA sequence $\mathbf{x}_i$ with L_i bases, we first pad the RNA sequence to L by appending the padding token 'PAD' to the end of $\mathbf{x}_i$. Then, we map each token in $\mathbf{x}_i$ to a number by using the following mapping:

$$\text{`A'} \mapsto 1, \text{`U'} \mapsto 2, \text{`C'} \mapsto 3, \text{`G'} \mapsto 4, \text{`PAD'} \mapsto 0. \tag{1}$$

We denote the sequence after padding and mapping by $\hat{\mathbf{x}}_i$, and we omit the subscript i in the following for brevity. We then transfer each number in $\hat{\mathbf{x}}$ to a vector by the input embedding along with the positional encoding. The input embedding matrix $E^{\text{in}} \in \mathbb{R}^{5 \times d_{\text{model}}}$ is a trainable lookup table, where d_{model} indicates the dimension of embeddings, and each row $E^{\text{in}}_{i,:}$ is an input embedding vector for the number i. Following [1], the positional encoding composed of sine and cosine functions is denoted by an $L \times d_{\text{model}}$ matrix E^{pos} such that

$$E^{\text{pos}}_{ij} = \begin{cases} \sin(i/10000^{j/d_{\text{model}}}) & \text{if } j \text{ is even,} \\ \cos(i/10000^{(j-1)/d_{\text{model}}}) & \text{if } j \text{ is odd.} \end{cases} \tag{2}$$

For the sequence $\hat{\mathbf{x}}$, after conducting the input embedding and adding the positional encoding, we obtain an $L \times d_{\text{model}}$ matrix X, whose i-th row $X_{i,:}$ is calculated by

$$X_{i,:} = E^{\text{in}}_{\hat{x}_i,:} + E^{\text{pos}}_{i,:}, \tag{3}$$

where $\hat{x}_i$ is the i-th number in the sequence $\hat{\mathbf{x}}$. The matrix X is used as the input of the Transformer encoder.

2.2 Transformer Encoder

We first use the Transformer encoder to generate a vector representation for each base in the RNA sequence. The Transformer encoder is composed of repetitive blocks, and each Transformer encoder block contains several attention heads.

We start with an introduction to the calculations within each attention head. Then, we explain the output of each Transformer encoder block based on the results of the attention heads. Finally, we describe the workflow of the whole Transformer encoder.

The j-th attention head in the i-th Transformer encoder block has three projection matrices $W^{ij}_Q \in \mathbb{R}^{d_{\text{model}} \times d_k}$, $W^{ij}_K \in \mathbb{R}^{d_{\text{model}} \times d_k}$, and $W^{ij}_V \in \mathbb{R}^{d_{\text{model}} \times d_v}$, where d_k and d_v are the dimensions of keys and values respectively. Given the input $I^i \in \mathbb{R}^{L \times d_{\text{model}}}$, this attention head first calculate the query matrix Q^{ij}, the key matrix K^{ij}, and the value matrix V^{ij} by

$$Q^{ij} = I^i W^{ij}_Q,\ K^{ij} = I^i W^{ij}_K,\ V^{ij} = I^i W^{ij}_V, \tag{4}$$

where $Q^{ij} \in \mathbb{R}^{L \times d_k}$, $K^{ij} \in \mathbb{R}^{L \times d_k}$, and $V^{ij} \in \mathbb{R}^{L \times d_v}$.

Then, this attention head computes the scaled dot-product attention based on Q^{ij}, K^{ij}, and V^{ij}. The result of the scaled dot-product attention of the j-th attention head in the i-th Transformer encoder block H^{ij} is calculated by

$$H^{ij} = \text{Attention}(Q^{ij}, K^{ij}, V^{ij}) = \text{Softmax}\left(\frac{Q^{ij} {K^{ij}}^{\mathrm{T}}}{\sqrt{d_k}}\right) V^{ij}, \tag{5}$$

where $H^{ij} \in \mathbb{R}^{L \times d_v}$.

Next, based on the attention results of the attention heads, we explain the output of the Transformer encoder block. The i-th Transformer encoder block contains an output projection matrix $W_O^i \in \mathbb{R}^{hd_v \times d_{\text{model}}}$. We denote the number of attention heads in the Transformer encoder block by h, and the attention results of the attention heads by $H^{i1}, H^{i2}, \ldots, H^{ih}$. Then, the result of the multi-head self-attention in the i-th Transformer encoder block denoted by $A^i \in \mathbb{R}^{L \times d_{\text{model}}}$ is obtained by

$$A^i = \left[H^{i1}, H^{i2}, \ldots, H^{ih}\right] W_O^i, \tag{6}$$

where $[\cdot]$ means the concatenation of matrices over the last dimension.

In the i-th Transformer encoder block, after computing A^i, we add the input matrix I^i to it to construct a residual connection and conduct the layer normalization [3]. The result $\widehat{A}^i$ after the transformations above is thereby denoted by

$$\widehat{A}^i = \text{LayerNorm}(A^i + I^i), \tag{7}$$

where "LayerNorm" indicates the layer normalization.

The result $\widehat{A}^i$ is then passed to a two-layer feed-forward network, and the computation is denoted by

$$F^i = \text{ReLU}(\widehat{A}^i W_1^i + b_1^i) W_2^i + b_2^i, \tag{8}$$

where $W_1^i \in \mathbb{R}^{d_{\text{model}} \times d_{\text{ff}}}$, $W_2^i \in \mathbb{R}^{d_{\text{ff}} \times d_{\text{model}}}$, $b_1^i \in \mathbb{R}^{d_{\text{ff}}}$, $b_2^i \in \mathbb{R}^{d_{\text{model}}}$, and d_{ff} denotes the dimension of the hidden layer.

Similar to Equation (7), we add $\widehat{A}^i$ to the output of the feed-forward network F^i and then conduct the layer normalization to obtain the final output of the i-th Transformer encoder block, i.e., the output denoted by $O^i \in \mathbb{R}^{L \times d_{\text{model}}}$ is calculated by

$$O^i = \text{LayerNorm}(F^i + \widehat{A}^i). \tag{9}$$

To summarize, if we denote the calculations from Equation (4) to Equation (6) by "MultiHeadSelfAttention", and denote the calculation of Equation (8) by "FeedForward", then calculations within the i-th Transformer encoder block are

$$\begin{aligned} A^i &= \text{MultiHeadSelfAttention}(I^i), \\ \widehat{A}^i &= \text{LayerNorm}(A^i + I^i), \\ F^i &= \text{FeedForward}(\widehat{A}^i), \\ O^i &= \text{LayerNorm}(F^i + \widehat{A}^i), \end{aligned} \tag{10}$$

where $I^i \in \mathbb{R}^{L \times d_{\text{model}}}$ and $O^i \in \mathbb{R}^{L \times d_{\text{model}}}$ indicate the input and the output of the i-th Transformer encoder block respectively. Figure 5 illustrates the i-th Transformer encoder block.

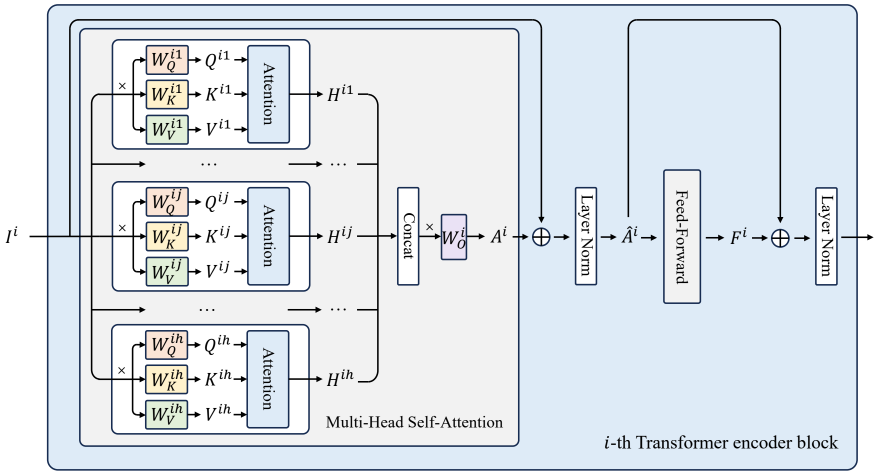

Supplementary Figure 5: Illustration of a Transformer encoder block

Next, we explain the workflow of the whole Transformer encoder. The Transformer encoder is composed of repetitive Transformer encoder blocks. The input of the first Transformer encoder block $I^1 = X$, where X is the input of the Transformer encoder obtained by Equation (3). The input of the i-th subsequent block $I^i = O^{i-1}$ for $i > 1$. The output of the Transformer encoder is the output of the last Transformer encoder block O^N, where N denotes the number of Transformer encoder blocks. We omit the superscript N and use O to indicate the output of the Transformer encoder for simplicity.

2.3 Pair-wise Concatenation

To further extract the relation between each pair of bases, we first conduct a pair-wise concatenation based on the output of the Transformer encoder O.

The result of pair-wise concatenation denoted by $C \in \mathbb{R}^{L \times L \times 2d_{\text{model}}}$ is computed as follows:

$$C_{i,j,:} = [O_{i,:}, O_{j,:}] \tag{11}$$

where $[\cdot, \cdot]$ indicates the concatenation over the last dimension. Figure 6 illustrates the pair-wise concatenation.

2.4 U-Net Encoder

The result of the pair-wise concatenation C is then passed to a series of U-Net convolutional layers for further processing. The workflow after the pair-wise concatenation is illustrated in Figure 7.

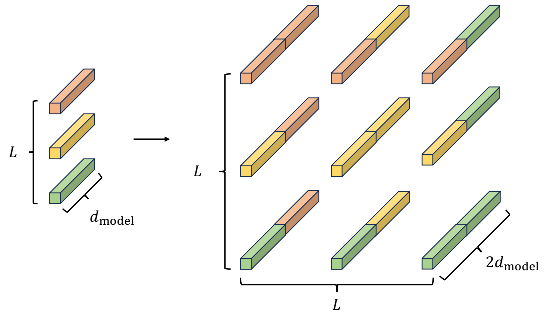

Supplementary Figure 6: Illustration of the pair-wise concatenation

Before yielding the $L \times L$ matrix prediction of the RNA secondary structure, we first pass C to the U-Net encoder composed of a stack of convolutional layers.

We first use two consecutive convolution layers sharing the same output channel number c_0^e to transform the $L \times L \times 2d_{\text{model}}$ input tensor C to an $L \times L \times c_0^e$ tensor. The subsequent layers could be divided into several similar blocks. The i-th block starts with a max-pooling layer with a 2×2 kernel that reduces the width of the feature map by half, i.e., $L_i^e = L_{i-1}^e/2$, where L_i^e is the width of the feature map in the i-th U-Net encoder block, and we set $L_0^e = L$. Then, it doubles the channel number by using two consecutive convolution layers sharing the same output channel number c_i^e such that $c_i^e = 2 \times c_{i-1}^e$. The output of the i-th block is thereby an $L_i^e \times L_i^e \times c_i^e$ tensor. The left side of Figure 7 illustrates the i-th U-Net encoder block.

Those blocks reduce the width of the feature map progressively, and we obtain a set of small feature maps after processing C by the U-Net encoder, which are then used to generate the $L \times L$ matrix prediction of the RNA secondary structure.

2.5 U-Net Decoder

The output of the U-Net encoder is then passed to the U-Net decoder, another stack of convolutional layers.

The i-th block first uses an up-sampling layer to double the width of the feature map, i.e., $L_i^d = 2 \times L_{i-1}^d$, where L_i^d is the width of the feature map of the i-th decoder block, and we set $L_0^d = L_{N^e}^e$, where N^e denotes the block number of the U-Net encoder. Then, it uses a convolutional layer to reduce the channel number to c_i^d such that $c_i^d = c_{i-1}^d/2$, and we set $c_0^d = c_{N^e}^e$. The up-sampling layer together with the convolutional layer above are usually called the "up-convolution", and the result after the up-convolution is an $L_i^d \times L_i^d \times c_i^d$ tensor. Then, we concatenate the output of the corresponding U-Net encoder block whose output channel number is equal to c_i^d with the result of the up-convolution over the last dimension to obtain an $L_i^d \times L_i^d \times 2c_i^d$ tensor. We further

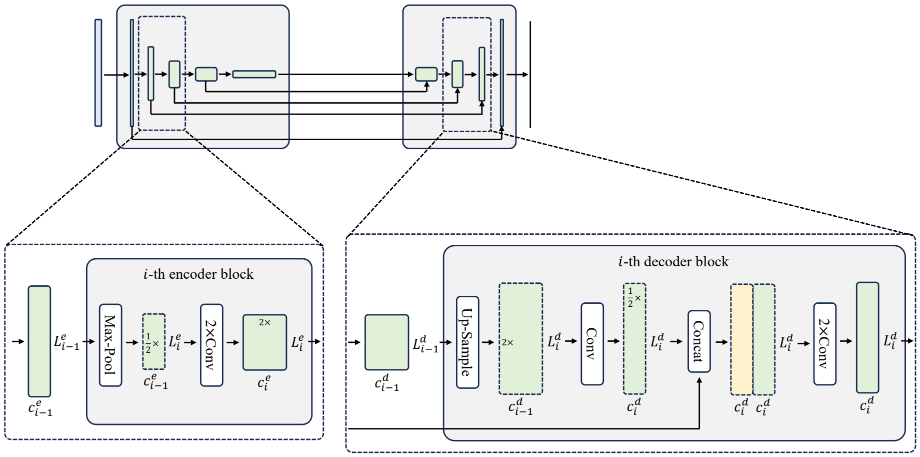

Supplementary Figure 7: Illustration of the U-Net encoder and decoder

pass this result to two consecutive convolutional layers sharing the same output channel number c_i^d, and the output of the i-th block is thereby an $L_i^d \times L_i^d \times c_i^d$ tensor. The right side of Figure 7 illustrates the i-th U-Net decoder block.

After processing the output of the encoder by the blocks above, the U-Net decoder uses a convolutional layer with output channel number 1 to produce an $L \times L \times 1$ tensor. By squeezing the last dimension, we obtain an $L \times L$ matrix M. Finally, we generate the symmetric output $\widehat{M}$ by averaging M and its transpose as follows:

$$\widehat{M} = \frac{M + M^{\mathrm{T}}}{2}. \tag{12}$$

3 Supplement: the Optimization of the Model

As we use an optimization target based on the cross-entropy, we start with a calculation of the gradients of logits when using softmax along with the cross-entropy, showing that those gradients are simple and meaningful. While training the model, we could consider either each row or each column of the prediction matrix and the label matrix as the prediction and the label of an L-class classification, and then optimize by rows or by columns. We could also take both cases into consideration and optimize by both rows and columns. Using the results of the calculation of the gradients above, we further compute the gradient of any entry M_{ij} in the matrix M, and we show those three kinds of optimizations are equivalent, and it is sufficient to optimize either by rows or by columns.

We use $\mathbf{z} \in \mathbb{R}^L$ denoted by

$$\mathbf{z} = [z_1, z_2, \ldots, z_L] \tag{13}$$

to indicate the logits produced by a model used for L-class classification. We

then use $\mathbf{p} \in \mathbb{R}^L$ to denote the prediction obtained by applying softmax to $\mathbf{z}$, and its c-th element p_c is calculated by

$$p_c = \frac{\exp(z_c)}{\sum_{i=1}^{L} \exp(z_i)}, \tag{14}$$

where $c \in \{1, 2, \dots, L\}$. We use $\mathbf{q} \in \mathbb{R}^L$ to represent the label denoted by

$$\mathbf{q} = [q_1, q_2, \dots, q_L], \tag{15}$$

where $\sum_{i=1}^{L} q_i = 1$. We define the cross-entropy loss $\ell_{\text{CE}}(\mathbf{p}, \mathbf{q})$ as

$$\ell_{\text{CE}}(\mathbf{p}, \mathbf{q}) = -\sum_{c=1}^{L} q_c \log(p_c). \tag{16}$$

In the following calculation, we use ℓ_{CE} to indicate $\ell_{\text{CE}}(\mathbf{p}, \mathbf{q})$ for brevity.

We have

$$\frac{\partial \ell_{\text{CE}}}{\partial p_{c^*}} = -\frac{q_{c^*}}{p_{c^*}} \tag{17}$$

for $c^* \in \{1, 2, \dots, L\}$. If $c^* = c$, then

$$\begin{aligned} \frac{\partial p_{c^*}}{\partial z_c} &= \frac{\partial p_c}{\partial z_c} \\ &= \frac{\exp(z_c)\left(\sum_{i=1}^{L} \exp(z_i) - \exp(z_c)\right)}{\left(\sum_{i=1}^{L} \exp(z_i)\right)^2} \\ &= p_c(1 - p_c). \end{aligned} \tag{18}$$

If $c^* \neq c$, then

$$\begin{aligned} \frac{\partial p_{c^*}}{\partial z_c} &= \frac{-\exp(z_{c^*})\exp(z_c)}{\left(\sum_{i=1}^{L} \exp(z_i)\right)^2} \\ &= -p_c p_{c^*}. \end{aligned} \tag{19}$$

We denote the Kronecker delta by δ defined as

$$\delta_{ij} = \begin{cases} 1 & \text{if } i = j, \\ 0 & \text{otherwise.} \end{cases} \tag{20}$$

Using the Kronecker delta, we combine the results of Equation 18 and Equation 19 as follows:

$$\frac{\partial p_{c^*}}{\partial z_c} = p_{c^*}(\delta_{cc^*} - p_c). \tag{21}$$

Then, we have

$$
\begin{aligned}
\frac{\partial \ell_{\mathrm{CE}}}{\partial z_c} &= \sum_{c^*=1}^{L} \frac{\partial \ell_{\mathrm{CE}}}{\partial p_{c^*}} \frac{\partial p_{c^*}}{\partial z_c} \\
&= -\sum_{c^*=1}^{L} \frac{q_{c^*}}{p_{c^*}} p_{c^*} (\delta_{cc^*} - p_c) \\
&= -\sum_{c^*=1}^{L} q_{c^*} \delta_{cc^*} + \sum_{c^*=1}^{L} q_{c^*} p_c \\
&= p_c - q_c.
\end{aligned}
\tag{22}
$$

To summarize, the calculations above show that

$$
\frac{\partial \ell_{\mathrm{CE}}}{\partial z_c} = p_c - q_c, \tag{23}
$$

and the gradients of the logits when using softmax along with the cross-entropy loss are the differences between the prediction and the label, which are simple and meaningful.

Based on the calculations above, we then show that it is equivalent to optimize the model by rows, by columns, and by both rows and columns of the prediction matrix and the label matrix.

We denote the direct output of the model by an $L \times L$ matrix M, the final output by $\widehat{M} = (M + M^{\mathrm{T}})/2$, and the modified label of the training sample by $\widehat{S}$. Then, we use P^r and P^c to denote the results of applying softmax on each row and each column of $\widehat{M}$ respectively.

In the following calculations, we show that the gradients of any entry M_{ij} when using softmax along with the cross-entropy loss are the same when we optimize the model by rows, by columns, and by both rows and columns. We omit the subscript "CE" and use ℓ to indicate ℓ_{CE} for simplicity.

We denote the optimization targets when we optimize by row, by column, and by both row and column by

$$
\ell^r = \frac{1}{L} \sum_{i=1}^{L} \ell \left(P^r_{i,:}, \widehat{S}_{i,:} \right), \ell^c = \frac{1}{L} \sum_{j=1}^{L} \ell \left(P^c_{:,j}, \widehat{S}_{:,j} \right), \ell^{rc} = \frac{1}{2} (\ell^r + \ell^c) \tag{24}
$$

respectively. Using Equation 23, we have

$$
\frac{\partial \ell^r}{\partial \widehat{M}_{ij}} = \frac{1}{L} \left(P^r_{ij} - \widehat{S}_{ij} \right), \frac{\partial \ell^c}{\partial \widehat{M}_{ij}} = \frac{1}{L} \left(P^c_{ij} - \widehat{S}_{ij} \right). \tag{25}
$$

According to the definition of $\widehat{M}$, we have

$$
\widehat{M}_{ij} = \frac{1}{2}(M_{ij} + M_{ji}), \widehat{M}_{ji} = \frac{1}{2}(M_{ji} + M_{ij}). \tag{26}
$$

Therefore, the gradients of M_{ij} are

$$\begin{aligned}
\frac{\partial \ell^r}{\partial M_{ij}} &= \frac{\partial \ell^r}{\partial \widehat{M}_{ij}}\frac{\partial \widehat{M}_{ij}}{\partial M_{ij}} + \frac{\partial \ell^r}{\partial \widehat{M}_{ji}}\frac{\partial \widehat{M}_{ji}}{\partial M_{ij}} = \frac{1}{2L}(P^r_{ij} - \widehat{S}_{ij}) + \frac{1}{2L}(P^r_{ji} - \widehat{S}_{ji}) \\
\frac{\partial \ell^c}{\partial M_{ij}} &= \frac{\partial \ell^c}{\partial \widehat{M}_{ij}}\frac{\partial \widehat{M}_{ij}}{\partial M_{ij}} + \frac{\partial \ell^c}{\partial \widehat{M}_{ji}}\frac{\partial \widehat{M}_{ji}}{\partial M_{ij}} = \frac{1}{2L}(P^c_{ij} - \widehat{S}_{ij}) + \frac{1}{2L}(P^c_{ji} - \widehat{S}_{ji}) \\
\frac{\partial \ell^{rc}}{\partial M_{ij}} &= \frac{\partial \ell^{rc}}{\partial \ell^r}\frac{\partial \ell^r}{\partial M_{ij}} + \frac{\partial \ell^{rc}}{\partial \ell^c}\frac{\partial \ell^c}{\partial M_{ij}} = \frac{1}{4L}(P^r_{ij} - \widehat{S}_{ij}) + \frac{1}{4L}(P^r_{ji} - \widehat{S}_{ji}) \\
&\qquad + \frac{1}{4L}(P^c_{ij} - \widehat{S}_{ij}) + \frac{1}{4L}(P^c_{ji} - \widehat{S}_{ji})
\end{aligned} \tag{27}$$

respectively. As $\widehat{M}$ is symmetric according to its definition,

$$P^r_{ij} = \frac{\exp(\widehat{M}_{ij})}{\sum_k \exp(\widehat{M}_{ik})} = \frac{\exp(\widehat{M}_{ji})}{\sum_k \exp(\widehat{M}_{ki})} = P^c_{ji} \tag{28}$$

holds. In other words, $P^r = (P^c)^{\mathrm{T}}$. Then, we use P^r_{ij} and P^r_{ji} to substitute P^c_{ji} and P^c_{ij} in Equation 27 respectively, and we have

$$\frac{\partial \ell^r}{\partial M_{ij}} = \frac{\partial \ell^c}{\partial M_{ij}} = \frac{\partial \ell^{rc}}{\partial M_{ij}} = \frac{1}{2L}P^r_{ij} - \frac{1}{2L}\widehat{S}_{ij} + \frac{1}{2L}P^r_{ji} - \frac{1}{2L}\widehat{S}_{ji}. \tag{29}$$

Therefore, no matter we optimize by rows, by columns, or by both rows and columns, the gradients of any entry M_{ij} in matrix M are identical, and this suggests the equivalence of the three optimization targets. As a result, instead of optimizing by both rows and columns, it is sufficient to optimize by either rows or columns.

Next, we explain the optimization target used in our training method. In the following discussion, we call each row (or column) of the prediction matrix a "sub-prediction", each row (or column) of the label matrix a "sub-label", and each pair of a base and its corresponding sub-label a "sub-sample".

In the calculations above, the loss of each sample ℓ^r (or ℓ^c) is defined as the average cross-entropy of each pair of sub-prediction and sub-label. If we take the whole batch into consideration, the loss of each batch could be further defined as the average of the loss of each sample. However, the number of bases that form base pairs and the number of bases that do not form base pairs are often unbalanced, and the number of bases that do not form base pairs is usually much larger, especially taking the padding tokens into consideration. Therefore, we introduce a weight for each cross-entropy to alleviate this issue, and we use the weighted average cross-entropy as the final optimization target.

We denote the shape of the prediction of a batch of RNA sequences by (b, L, L), where b is the size of this batch. In each batch, the number of sub-samples is bL. We denote the cross-entropy of the i-th sub-sample by ℓ_i, where $i \in \{1, 2, \ldots, bL\}$. Then, we denote the set of the indices of sub-samples whose

bases do not form base pairs by $\mathbf{D}$, and the size of $\mathbf{D}$ by $|\mathbf{D}|$. The optimization target ℓ is thereby calculated by

$$\ell = \frac{1}{bL}\left(\sum_{i \notin \mathbf{D}} \ell_i + \frac{bL - |\mathbf{D}|}{|\mathbf{D}|}\sum_{i \in \mathbf{D}} \ell_i\right). \tag{30}$$

It is trivial to show the equivalence of the three optimization targets above after introducing a weight for each sub-sample. For simplicity, we denote the weight for sub-samples whose indices are not in $\mathbf{D}$ by w_1, and the weight for other sub-samples by w_2, such that

$$w_1 = \frac{1}{bL},\ w_2 = \frac{bL - |\mathbf{D}|}{bL|\mathbf{D}|}. \tag{31}$$

We now go back to Equation 27 and consider the loss of each sample for simplicity. We denote the weighted optimization targets by $\hat{\ell}^r$, $\hat{\ell}^c$, and $\hat{\ell}^{rc}$ respectively. After introducing those weights, we have

$$\begin{aligned}
\frac{\partial \hat{\ell}^r}{\partial M_{ij}} &= \frac{1}{2w_a}(P^r_{ij} - \widehat{S}_{ij}) + \frac{1}{2w_b}(P^r_{ji} - \widehat{S}_{ji}) \\
\frac{\partial \hat{\ell}^c}{\partial M_{ij}} &= \frac{1}{2w_b}(P^c_{ij} - \widehat{S}_{ij}) + \frac{1}{2w_a}(P^c_{ji} - \widehat{S}_{ji}) \\
\frac{\partial \hat{\ell}^{rc}}{\partial M_{ij}} &= \frac{1}{4w_a}(P^r_{ij} - \widehat{S}_{ij}) + \frac{1}{4w_b}(P^r_{ji} - \widehat{S}_{ji}) \\
&\quad + \frac{1}{4w_b}(P^c_{ij} - \widehat{S}_{ij}) + \frac{1}{4w_a}(P^c_{ji} - \widehat{S}_{ji})
\end{aligned} \tag{32}$$

where $w_a, w_b \in \{w_1, w_2\}$, because the i-th row of $\widehat{S}$ and the i-th column of $\widehat{S}$ are exactly the same sub-label for the same base, as $\widehat{S}$ is symmetric. Using Equation 28, we then substitute P^c_{ij} and P^c_{ji} in the equations above to P^r_{ji} and P^r_{ij} respectively, and it is trivial to show that

$$\frac{\partial \hat{\ell}^r}{\partial M_{ij}} = \frac{\partial \hat{\ell}^c}{\partial M_{ij}} = \frac{\partial \hat{\ell}^{rc}}{\partial M_{ij}} \tag{33}$$

still holds.

References

[1] Ashish Vaswani, Noam Shazeer, Niki Parmar, Jakob Uszkoreit, Llion Jones, Aidan N Gomez, Łukasz Kaiser, and Illia Polosukhin. Attention is all you need. *Advances in neural information processing systems*, 30, 2017.

[2] Olaf Ronneberger, Philipp Fischer, and Thomas Brox. U-net: Convolutional networks for biomedical image segmentation. In *Medical image computing and computer-assisted intervention–MICCAI 2015: 18th international*

conference, Munich, Germany, October 5-9, 2015, proceedings, part III 18, pages 234–241. Springer, 2015.

[3] Jimmy Lei Ba, Jamie Ryan Kiros, and Geoffrey E Hinton. Layer normalization. *arXiv preprint arXiv:1607.06450*, 2016.
